# Pulsed Field Ablation of Superior Vena Cava: Feasibility and Safety of Pulsed Field Ablation

**DOI:** 10.3389/fcvm.2021.698716

**Published:** 2021-08-09

**Authors:** Tongjian Zhu, Zhen Wang, Songyun Wang, Tiancai Shi, Xiaolin Zhu, Kezhong Ma, Zhuo Wang, Jinnian Gao, Hong Jiang

**Affiliations:** ^1^Department of Cardiology, Renmin Hospital of Wuhan University, Wuhan, China; ^2^Sichuan Jinjiang Electronic Technology Co. Ltd., Sichuan, China; ^3^Department of Cardiology, Xiangyang Central Hospital, Affiliated Hospital of Hubei University of Arts and Science, Xiangyang, China

**Keywords:** pulsed field ablation, superior vena cava, arrhythmias, phrenic palsy, sinus node injury

## Abstract

**Background:** Studies have shown that pulsed field ablation (PFA) has excellent effectiveness and safety in pulmonary vein isolation (PVI). However, there are few reports about the application of PFA, especially the alternating current (AC) biphase PFA, in superior vena cava (SVC) isolation, and its effectiveness and safety are still unclear.

**Objective:** To investigate the efficacy and safety of the AC biphase PFA for SVC isolation, and to provide evidence for the clinical use of PFA for SVC.

**Methods:** Eight pigs and two dogs were included in the study. PFA was delivered to these pigs and dogs. Pacing threshold and electrogram data were recorded before and after PFA. Voltage mapping of SCV was obtained before, after, and 3 weeks after PFA. At the end, all animals were euthanatized for gross pathology analysis.

**Results:** For eight pigs, the median pacing threshold was 1.5 (1.4, 2.75) mA before PFA, while > 6.0 mA after PFA for all animals. The average electrogram amplitude reduction was 61.33 ± 24.90% for ablations with the initial amplitude≥0.5 mv. For two dogs, pacing threshold change and electrogram amplitude reduction were also observed. No phrenic palsy or sinus node injury was observed during PFA in any animal. Furthermore, voltage mapping showed that the voltage amplitude was significantly decreased in all animals and this could be kept for more than 3 weeks. Moreover, transmural tissue damage with reserved vessel and nerve were shown, no SVC stenosis was found at 3 weeks after PFA.

**Conclusion:** PFA can effectively isolate SVC. Transmural tissue damage of SVC can be achieved without phrenic palsy, sinus node injury nor SVC stenosis.

## Introduction

Pulmonary vein isolation (PVI) is the basis of catheter ablation for atrial fibrillation (AF) (1). Although, with the continuous improvement of catheter ablation methods and techniques, the effectiveness and safety of PVI are also improving, the recurrence of AF is still very common during long-term follow-up (2, 3). Some patients with AF recurrence may be related to non-pulmonary vein-derived triggers (4), and the superior vena cava (SVC) is one of the most common non-pulmonary vein triggers (5). Many studies have demonstrated that ablation and isolation of SVC (SVCI) triggers can improve the long-term maintenance of sinus rhythm after AF ablation (6). At present, SVCI is mainly performed by point-by-point radiofrequency catheter ablation in clinical practice. The ablation process may lead to sinoatrial node injury, phrenic nerve paralysis, and SVC stenosis and other related complications, which has certain limitations (7–9).

Pulsed field ablation (PFA), also known as irreversible electroporation, is a new kind of energy ablation, which can achieve the purpose of non-thermal ablation through the formation of irreversible micropores in the cell membrane by instantaneous discharge, resulting in cell apoptosis (10). Compared with radiofrequency ablation (RFA), PFA is characterized by stronger tissue selectivity and shorter ablation time (a few seconds), which can damage myocardial tissue without heating the tissue and protect the surrounding critical structures, effectively reducing perioperative complications and shortening operative time (11, 12). Recently, a number of animal and clinical studies (13, 14) have shown that PFA has excellent effectiveness and safety in PVI. It is less likely to cause complications such as esophageal injury, phrenic nerve injury, and pulmonary vein stenosis. It is particularly attractive for catheter ablation of AF. Therefore, the isolation of SVC by PFA may theoretically reduce the incidence of complications. However, there are few reports on the application of PFA in SVCI (15, 16), and the total number of animal models in related researches is not much. So, its effectiveness and safety are still unclear. What's more, the catheter used in this study is a self-developed seven-electrode circular array PFA catheter, which is improved on the Lasso electrode used for pulmonary vein mapping. It can not only transmit alternating current (AC) bipolar pulse, but also be used as pacing and mapping electrode. Theoretically, compared with the previous unipolar PFA, it has the advantages of stronger tissue targeting, no need for general anesthesia, and minimal muscle contraction. This study intends to isolate pig's and dog's SVC through AC biphasic PFA waveform and analyze the electrophysiological and histopathological characteristics of SVC before and after ablation to explore its effectiveness and safety.

## Methods

### Animal Preparation

Eight pigs (five female and three male, 3.5–5.5 months, 39–51 kg) and two canines (male, 12–18 months, 26–40 kg) were included in this study. The experimental protocol was approved by the Technical Committee on laboratory animal standardization of Sichuan jinjiang Electronic Technology Co. Ltd under approval number JJJS20003 and followed the NIH guideline of Care and Use of Laboratory Animals. All surgeries were performed under 3% sodium pentobarbital anesthesia. The initial dose was set at 1 ml/kg and the maintenance dose at 0.2 ml/h/kg. During the procedure, the body surface electrocardiogram was recorded with a computer-based Lab System (Lead 7000, Sichuan Jingjiang Electronic Technology, China).

### Mapping, Ablation, and Pacing Capture

**Figure 1:**
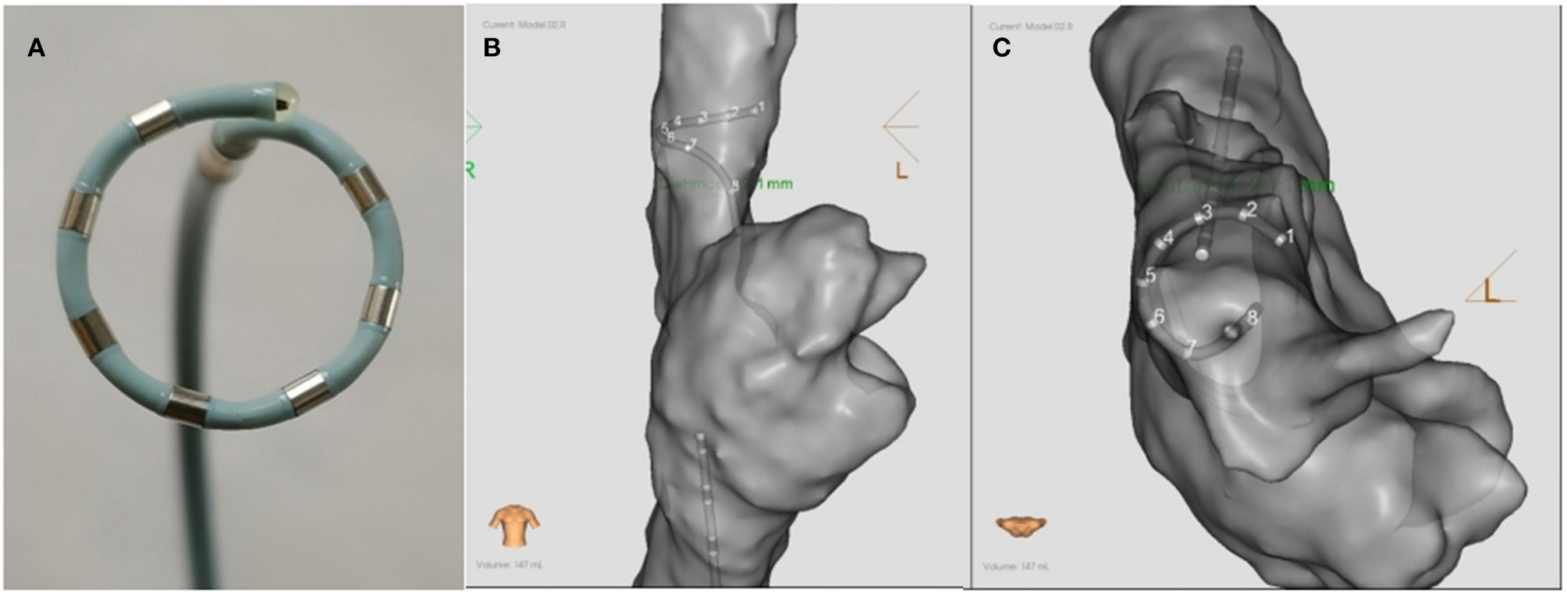
The schematic diagram **(A**) and the ablation positions **(B,C)** of the PFA catheter. **(A)** The catheter was an alternating current, bipolar, multi-electrode circular 6F catheter with seven electrodes. The diameter of the loop was 15 mm, and the inter-electrode distance was 4 mm. **(B,C)** show the ablation postions of PFA in the SVC in the anterior-posterior and superior position, respectively. PFA, Pulsed field ablation.

After anesthesia and skin preparation, the animals were supine on the animal experimental platform, and their limbs were connected with ECG leads. The reference electrode was pasted on the body surface outside the thorax as a reference for three-dimensional system positioning. Two 8.5-F fixed-curve sheath (St Jude, USA) was placed in the left and right femoral vein. Heparin was administered to keep the activated clotting time 350–400 s. A seven-electrode circular array PFA catheter (Figure 1, AC bipolar PFA, 15 mm loop, 6F conduit, seven electrodes with electrode spacing of 4 mm; PFA8D15LT, Sichuan Jingjiang Electronic Technology, China) and a CS catheter (D6S10282L, Sichuan Jingjiang Electronic Technology, China) were placed through the right femoral vein to the SVC. A contact force irrigated RF catheter (FT8D06LTC, Sichuan Jingjiang Electronic Technology, China) was placed through the left femoral vein to the atria. Right atria anatomical mapping and SVC anatomical and voltage mapping was conducted with PFA catheter and the RF catheter before and after each PFA. The operation is completed under the 3D mapping system with magnetic positioning function and assisted by X-ray. Biphasic PFA applications wasdelivered to ablate SVC with the PFA catheter (PFA8D15LT, Sichuan Jingjiang Electronic Technology, China) and PFA generator (LEAD-PFA, Sichuan Jingjiang Electronic Technology, China). The voltage amplitude is ±1,000 V in dogs and ±1,500 V in pigs. To verify the effect of PFA, exit block was tested by PFA catheter pacing at SVC and observing the pacing capture of the RF catheter in the atria. Voltage mapping before and after ablation was also used to verify the PFA effect. Once all ablations were completed, pigs or dogs were recovered under veterinary supervision.

### Electrogram Data Collecting and Processing

Electrogram was recorded during the whole operation, and intracardiac electrogram (EGM) was recorded on the LEAD-Mapping D system (Sichuan Jingjiang Electronic Technology, China) from six electrode pairs on the circular array catheter (E1-2, E2-3, E3-4, E4-5, E5-6, and E6-7). The pre-ablation electrogram amplitude < 0.5 mv or without voltage amplitude change after PFA was indicated poor contact with non-ablated tissue, and the data of which ablation was not included in the statistics (14). As compared to the pre-ablation, the percent reduction in EGM amplitude of post-ablation was calculated (15).

### Gross Pathology and HE Staining

Three weeks after ablation, each experimental model animal was euthanized and the hearts were then submitted for histopathologic examination. The ablated SVC were examined before and after hematoxylin eosin (HE) staining. The specimens were dehydrated, paraffin embedded and sectioned about 4 μm. After slides were stained with HE, the pathological changes of vein sections, edema and thrombosis were evaluated under microscope (15).

### Statistical Analysis

Continuous variables are expressed as mean ± SD or median (range) and categorical variables are given as count and percentage. Continuous variables were compared between the groups *via* the Mann-Whitney U test or Student's *t*-test and categorical variables were compared by chi-square analysis or Fisher's test, as appropriate. A *P*-value < 0.05 was considered significant. Statistical analyses were performed with SPSS 26.0 software (SPSS Inc., Chicago, IL, USA).

## Results

### Pulsed Field Applications and Dosages

In this study, two canines and eight pigs were included. PFA was delivered to the dogs and the pigs. As for pigs, 6 (5.25, 8.5) applications of PFA was delivered to each. And the median dosage for each application was 2.25 (1.8, 3.15) ms. (Table 1). For two dogs, 11 and 14 applications of PFA were delivered, respectively. In total, one dog received 3.2 ms PFA and the other 4.8 ms PFA.

**Table 1:** Pulsed field applications and dosages.

**Animal**	**Animal number**	**Application (times)**	**Dosage (ms)**
Pig	a	23	3.2
	b	9	5.2
	c	5	3
	d	5	1.7
	e	7	2.7
	f	6	1.8
	g	6	1.8
	h	6	1.8
	Median	6 (5.25, 8.5)	2.25 (1.8, 3.15)
Dog	a	14	3.2
	b	11	4.8

### EGM and Voltage Mapping

**Figure 2:**
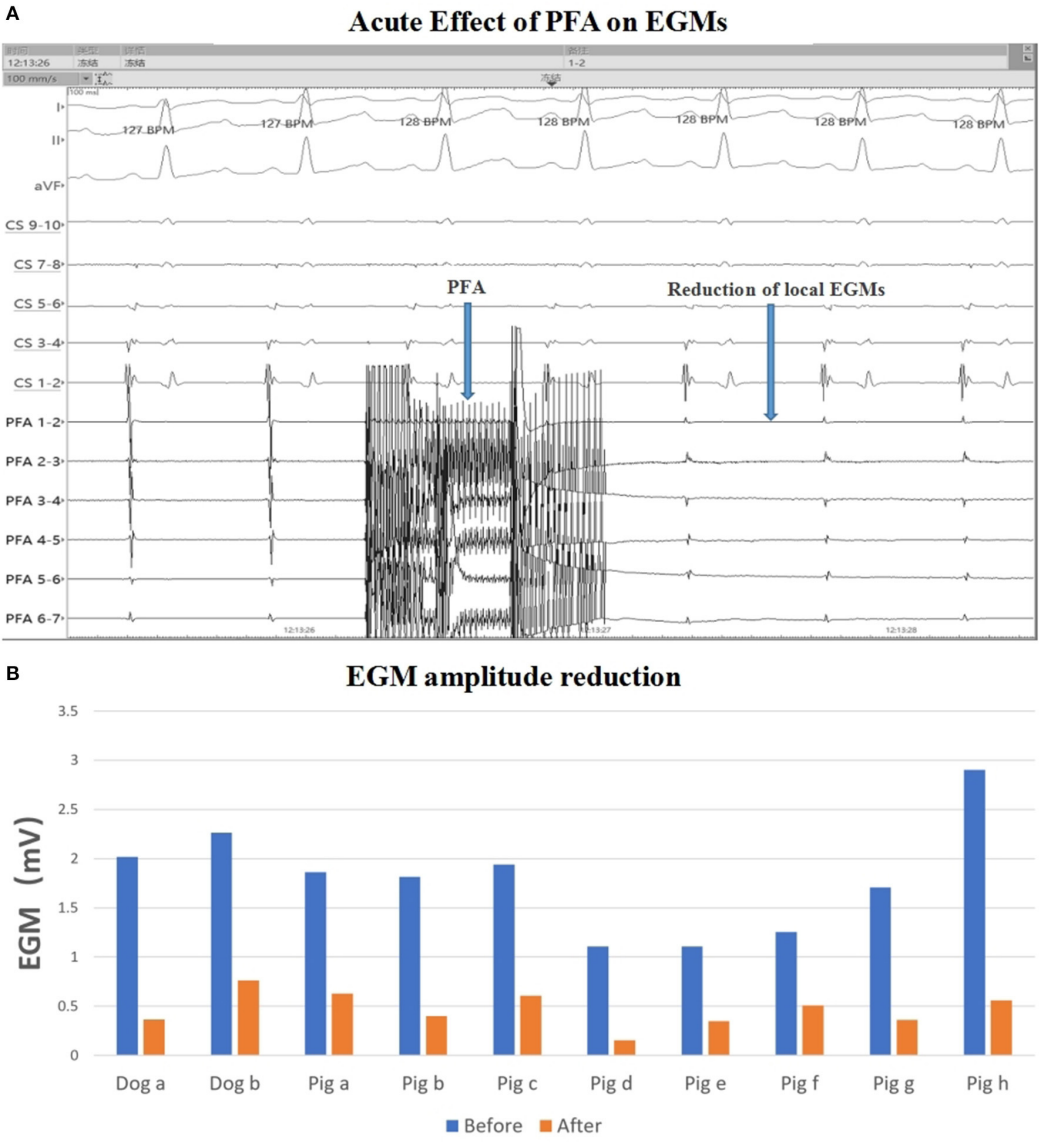
EGM amplitude change. **(A)** EGM signal reduction with PFA; **(B)** EGM amplitude before and after ablation in each animal. The mean EGM amplitude were reduced after ablation. PFA, Pulsed field ablation.

For eight pigs, 67 ablations were performed and 402 EGMs were recorded. In which, the applications with voltage amplitude < 0.5 mv before PFA or without voltage amplitude change after PFA, which represented bad tissue contact in the targeted area, were excluded. Finally, 206 (51.24%) EGMs were included in the statistics. For the applications with good contact, the EGM amplitude were reduced (Figure 2), the mean reduction was 61.33% ± 24.90% (Table 2), and the applications with EGM amplitude loss >50% were 145 (70.39%).

**Table 2:** Acute electrical results.

**Animal**	**Animal** ** number**	**Pacing threshold (mA)**		**EGM amplitude** reduction^*^
		**Pre-PFA**	**Post-PFA**	
Pig	a	2.0	>6.0	55.72%
	b	1.2	>6.0	67.47%
	c	3.0	>6.0	50.65%
	d	1.4	>6.0	78.83%
	e	1.4	>6.0	62.00%
	f	1.4	>6.0	53.05%
	g	1.6	>6.0	69.40%
	h	4	>6.0	71.33%
	Median/Mean	1.5 (1.4, 2.75)	>6.0	61.33% ± 24.90%
Dog	a	1.8	>6.0	75.01%
	b	0.6	>6.0	51.13%

* *The ablation records with voltage amplitude < 0.5 mv before PFA or without voltage amplitude change before and after PFA were excluded*.

*PFA, pulsed field ablation*.

For two dogs, 150 EGMs were recorded after 25 ablations. Ninety-five (63.33%) EGMs were included in the statistics and the mean reduction of EGM amplitude was 61.68 ± 24.20%, and the applications with EGM amplitude loss >50% were 61 (64.21%).

**Figure 3:**
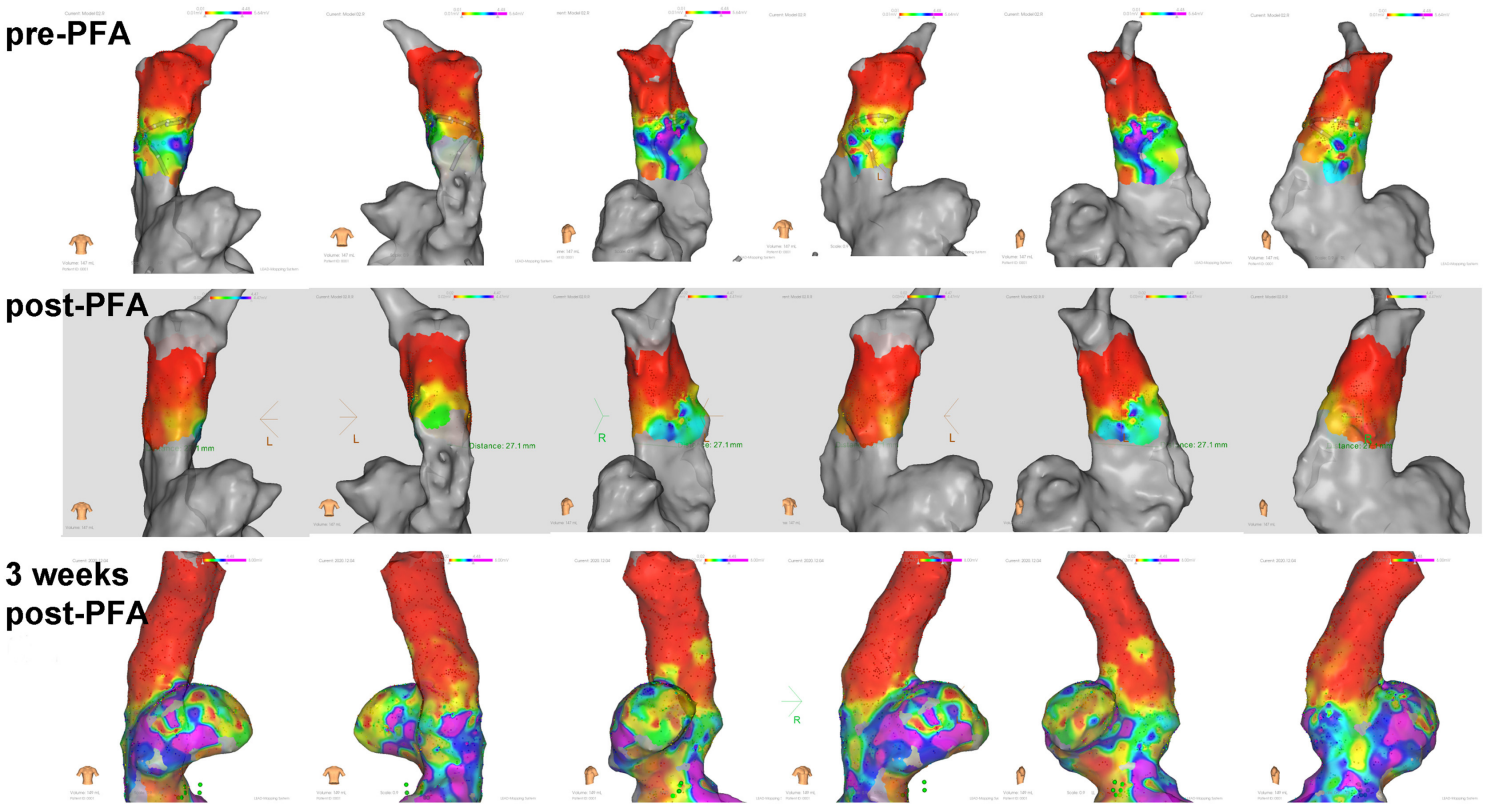
Voltage mapping of superior vena cava in different body positions. Potential reduction was observed after ablation and during 3 weeks follow up.

Consistently, the voltage mapping also showed that the potential was significantly reduced after PFA in all animals. Furthermore, 3 weeks latter, potential reduction were still observed (Figure 3).

### Pacing Capture and Complications

**Figure 4:**
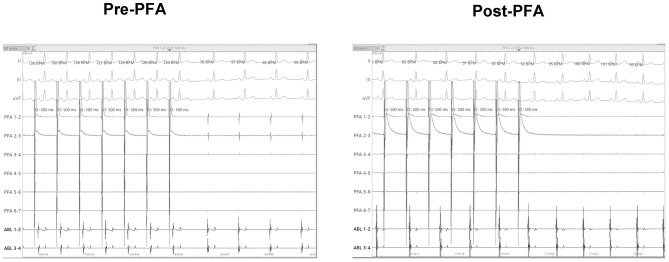
Pacing threshold measuring. Before ablation, catheter in superior vena cava can record atrial potential and successfully pace the atrium. After ablation, PFA catheter in superior vena cava could not record the atrial electrical activity and pacing the atrium. PFA, Pulsed field ablation.

Before PFA, the median pacing threshold was 1.5 (1.4, 2.75) mA in pigs, the pacing threshold of dogs was 1.8 mA and 0.6 mA, respectively. After PFA, The median pacing threshold in all animals was >6.0 mA (Table 2). Capture loss was observed in all animals after ablation (Figure 4). What's more, after ablation, the PFA catheter located in superior vena cava did not record atrial electrical activity (Figure 4), which means atrial electrical activity could not be conducted to superior vena cava. No phrenic palsy or sinus node injury was observed during PFA in any animal.

### Gross Pathology and HE Staining

**Figure 5:**
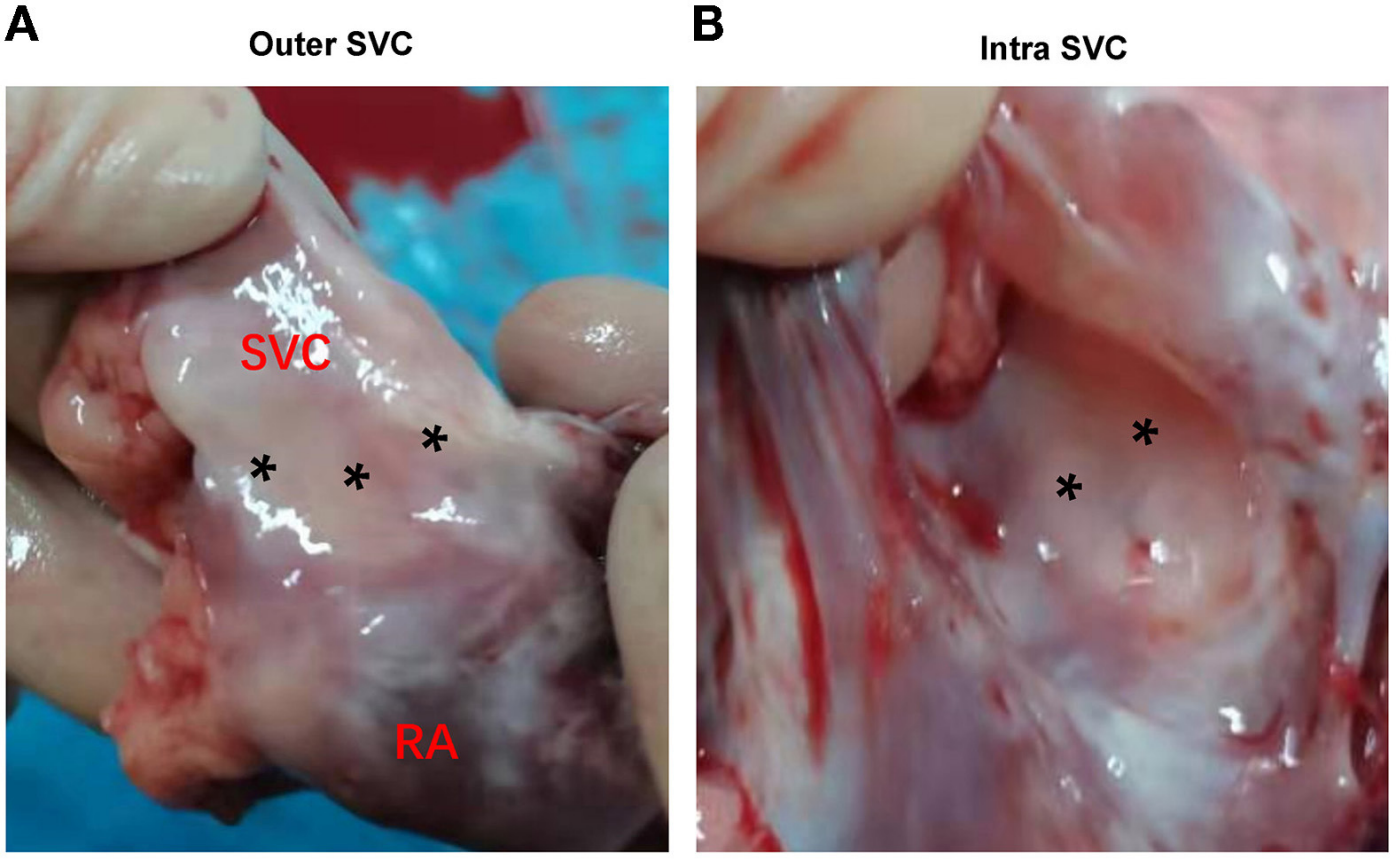
Gross pathology. **(A)** Outer aspect of transmural lesions in the SVC. **(B)** Intra aspect of the same lesions in SVC. SVC, superior vena cava. Asterisks indicate the ablation area.

**Figure 6:**
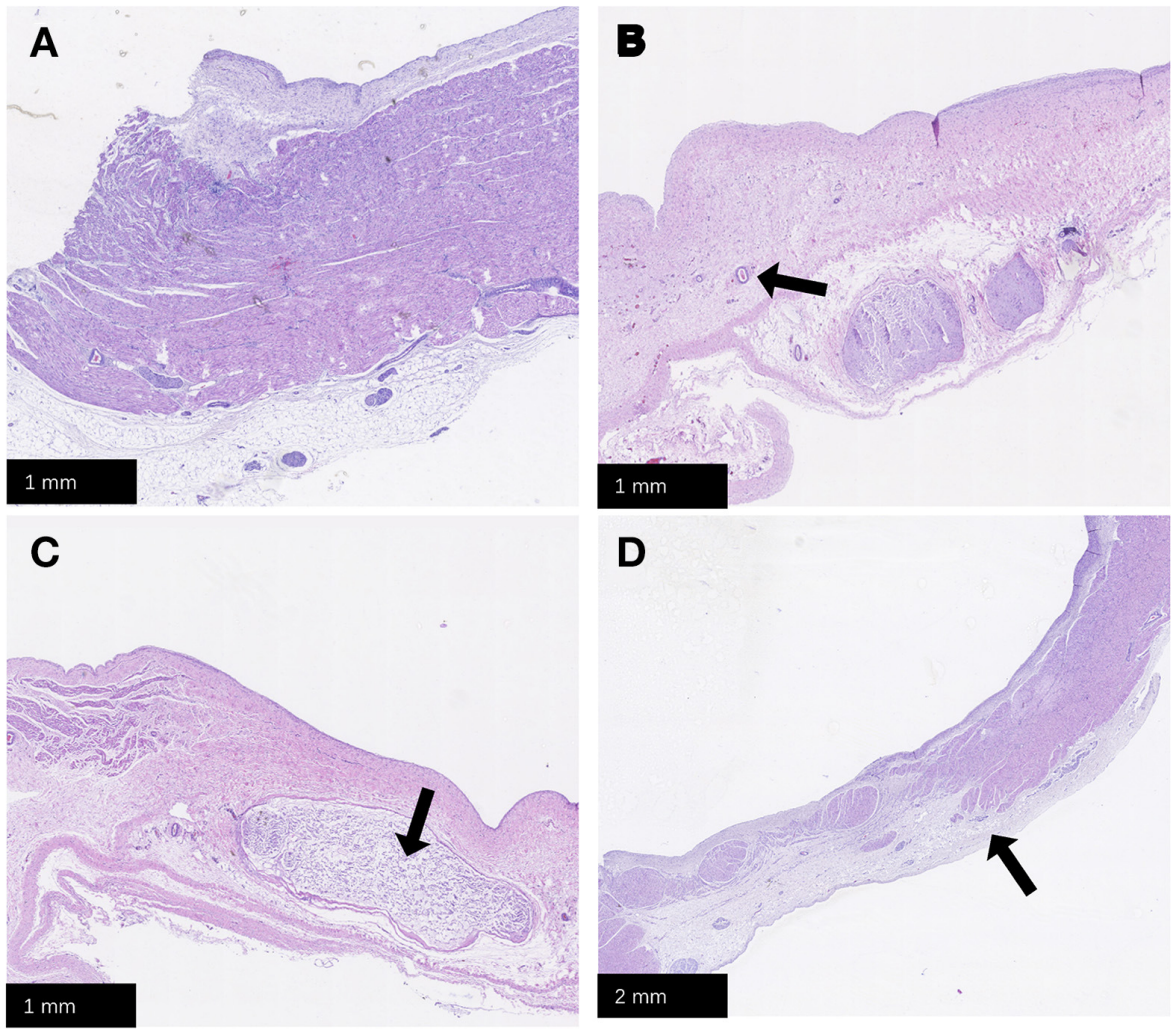
HE staining. **(A)** Normal SVC tissue; **(B)** Transmural damage caused by PFA, the arrow points to the reserved vascular structure; **(C)** The arrow points to the reserved nerve tissue; **(D)** The arrow points to the position of edge of PFA damage. PFA, pulsed field ablation. SVC, superior vena cava.

No animal died during the follow-up period. Three weeks after ablation, the animals were dissected to check the tissue condition of the SVC in the ablation area. The intimal surface was smooth and intact. No SVC stenosis was found. There were no signs of endometrial ablation, redness, and protein degeneration (Figure 5). HE staining demonstrated the edge was obvious. What's more, transmural ablation damage occurred in the ablation area, the endocardium, myocardium, and epicardium were loose and unevenly stained, and a large number of muscle fibers were broken and dissolved. The morphology and structure of blood vessels and nerve cells were reserved (Figure 6).

## Discussion

In this study, the feasibility of SVCI by PFA with seven-electrode circular AC bipolar PFA catheter was proved in pig and dog experiments. The amplitude and stimulation threshold of the local SVC potential were measured using a mapping catheter at multiple locations of the SVC before and after ablation, and 3 weeks after ablation. The SVC was then sliced, stained, and histologically examined at 3 weeks after ablation. The results showed that the immediate potential amplitude of SVC was significantly reduced after PFA, and further, potential reduction was observed during the 3-week follow-up. In addition, the stimulation threshold of SVC was significantly increased after ablation. Histological results after 3 weeks showed that most of the ablation sites could achieve transmural injury, and no stenosis was found in SVC. No complications such as sinoatrial node injury and phrenic nerve injury were observed in the immediate and 3-weeks follow-up after PFA of SVC.

Although, PVI remains the cornerstone of catheter ablation for the elimination of AF triggers currently, many studies have reported the presence of non-pulmonary vein origin foci in some patients with paroxysmal or persistent AF in recent years. Furthermore, in patients with high risk factors (e.g., older age, female, obesity, significant atrial remodeling and scarring, sleep apnea, heart failure, valvular heart disease, and hypertrophic cardiomyopathy), the incidence of AF triggered by external pulmonary venous triggers appears to be higher. PVI alone might not be enough for these patients and ablation of non-pulmonary venous triggers, including SVC, was necessary (17–19). Non-pulmonary venous triggers can be induced by standardized induction protocols, usually from discontinuous anatomical areas, including the area around the mitral and tricuspid annulus, crista terminalis (CT), interatrial septum, left atrium posterior wall, SVC, coronary sinus (CS), and left atrial appendage (LAA), etc., (20). As one of the most common non-pulmonary vein triggers, the incidence of SVC is about 5.3 ~12.8% according to different literature reports (5, 8, 21). The right atrial cuff extends to the SVC and contains autonomously active cells, thus, SVC is also an important matrix for the occurrence of AF. Higuchi et al. (22) found that the length of the SVC muscle sleeve in patients with AF of SVC origin is longer than that in patients with non-SVC origin. Ejima et al. (23) showed that patients with PVI+ empirical SVCI significantly reduced the recurrence rate of AF after single ablation compared with patients with PVI alone.

Currently, SVCI is mainly performed point-to-point ablation through RFA to achieve circular isolation in clinical practice, which is not only time-consuming, but also has many complications. The PFA can selectively act on the myocardial cell membrane by releasing the non-thermal energy of high electric field in a very short time, resulting in irreversible electroporation, leakage of cell contents, destruction of intracellular homeostatic environment, and death of myocardial cells. At the same time, the inflammatory reaction is slight, and the influence on the surrounding tissues is minimal (10). Recently, a number of basic and clinical studies have confirmed that PFA can safely and effectively isolate the pulmonary veins (13, 14). Compared with RFA and cryo-balloon ablation (CBA), PFA may cause fewer complications of peripheral tissue injury such as pulmonary vein stenosis and phrenic nerve injury (24). Although, there is some anatomical similarity between SVC and pulmonary vein, there are few reports on the application of PFA in SVCI. The results of this study showed that PFA can safely and effectively isolate SVC. In this study, all animals achieved acute electrical isolation of SVC. During the mean follow-up observation of 3 weeks, no recovery of SVC potential occurred in all the isolated SVC, and no obvious complications were observed.

Due to the anatomical location of the SVC adjacent to the right phrenic nerve and the sinoatrial node, the most concerned complications during PFA in SVC are sinoatrial node injury, phrenic nerve injury and SVC stenosis. Previous studies have reported that the incidence of sinus node injury caused by RFA was 2.0~4.5% (25), and the incidence of phrenic nerve injury was 2.1% (8). The phrenic nerve is often accompanied by SVC, and the RFA catheter can be used to locate the phrenic nerve by high-precision mapping, thus, avoiding ablation at the site of phrenic nerve capture. However, this does not completely prevent phrenic nerve injury, or even effectively isolate SVC for fear of phrenic nerve injury. Many studies have reported that RFA leads to sinoatrial node injury, and the possible mechanism is the damage to the sinoatrial node artery and the inaccurate determination of sinoatrial node location (25). Severe stenosis of SVC caused by RFA has also been reported, which may be related to eschars and collagen contracture associated with the high temperature effects (9). Gianni et al. (26) recently demonstrated a novel segmental radiofrequency SVC isolation approach, which was accomplished by targeting the septal segment of SVC and sites with early activation in the posterior SVC-right atrial (RA) junction and RA posterior wall. This might eliminate the risk of sinoatrial node injury or SVC stenosis by preserving the lateral and anterior sides of the SVC. However, PN injury could not be completely excluded, and the long-term effect of its SVCI still needs further study to confirm. In present study, SVCI was performed in all animals, and no sinoatrial node injury or phrenic nerve injury occurred during acute and 3-week follow-up, and no SVC stenosis was found on histological examination after 3 weeks. It is shown that compared with RFA, electrical isolation of SVC by PFA may have better security.

PFA has a strong resistance organization specificity. Compared with tissues such as vessels, nerves and esophagus, myocardial cells have the lowest pulsed electric field threshold, and the sensitivity of cardiomyocytes to pulsed electric field ablation is much higher than that of other cell types, which means that the appropriate threshold voltage of pulsed electric field would preferentially damage the myocardium, and have little impact on the adjacent tissues (27). Stewart et al. (14) used a nine-electrode circulararray pulmonary vein ablation catheter (PVAC GOLD™, Medtronic) to perform intracardiac ablation in pigs, histological examination of myocardial tissue after 2 weeks showed that PFA caused death and fibrosis of myocardial cells without damaging myocardial tissue outside the effective ablation field. Compared with RFA, PFA showed more uniform myocardial fibrosis, less epicardial fat inflammation, and less vascular remodeling. Reddy et al. (13) reported the first acute clinical experience with pulsed electric field ablation of AF, PFA was used to complete the electrical isolation of circumferential pulmonary vein in 15 patients with AF. The results showed that PFA had short operation time, no complications such as acute pulmonary vein stenosis, recovery of pulmonary venous potential conduction, phrenic nerve injury, etc., and could quickly and effectively isolate the pulmonary vein for specific injury of myocardial tissue.

### Clinical Implication

PFA with the seven-electrode circular array PFA catheter can safely and effectively isolate SVC. The advantage of PFA is to shorten the ablation time of AF, reduce the difficulty of ablation, and reduce the probability of complications such as SVC stenosis, phrenic nerve palsy and sinoatrial node injury. It is easy to popularize and has the potential to become a new energy source for ablation of AF.

### Limitations

This study is an animal study. Although, pigs and canines are often used in the study of arrhythmia models, there are still some differences between the animal SVC anatomy and the human body, and its safety and effectiveness need to be further confirmed in human clinical studies. What's more, complications like phrenic nerve or sinoatrial node injury is a low frequency event and it may need hundreds or thousands of animals to see it happen. However, the number of animals used in this study is too small to explore the complications of the PFA. In the future, more studies with large size are needed.

### Conclusion

PFA with the seven-electrode circular array PFA catheter can effectively isolate SVC. Transmural tissue damage of SVC can be achieved without phrenic palsy, sinus node injury nor SVC stenosis.

## Data Availability Statement

The raw data supporting the conclusions of this article will be made available by the authors, without undue reservation.

## Ethics Statement

The animal study was reviewed and approved by Technical Committee on laboratory animal standardization of Sichuan Jinjiang Electronic Technology Co. Ltd.

## Author Contributions

HJ and JG: conceived and designed the study. TZ, ZW, and SW: data collection and analyzed the data. TS, XZ, and KM: quality control the study and revision. TZ and ZW: wrote the paper. The manuscript was approved by all above authors.

## Conflict of Interest

TS, XZ, and JG are employees of Sichuan jinjiang Electronic Technology Co. Ltd. The remaining authors declare that the research was conducted in the absence of any commercial or financial relationships that could be construed as a potential conflict of interest.

## Publisher's Note

All claims expressed in this article are solely those of the authors and do not necessarily represent those of their affiliated organizations, or those of the publisher, the editors and the reviewers. Any product that may be evaluated in this article, or claim that may be made by its manufacturer, is not guaranteed or endorsed by the publisher.
